# Palladium-Catalyzed C–H Arylation of Quinoidal Scaffolds Through Homogeneous and Heterogeneous Pathways: Advancing Toward Trypanocidal Prototypes

**DOI:** 10.1021/acsomega.5c05006

**Published:** 2025-08-14

**Authors:** José M. C. Tavares Junior, Eduardo F. S. Guimarães, Mateus P. Nunes, Renata G. Almeida, Maria H. Araujo, Victor F. S. Ramos, Rubem F. S. Menna-Barreto, Joel A. Tchuiteng Kouatchou, Edmond Gravel, Eric Doris, Guilherme A. M. Jardim, Eufrânio N. da Silva Júnior

**Affiliations:** † Departamento de Química, Instituto de Ciências Exatas, 28114 Universidade Federal de Minas Gerais, Belo Horizonte, Minas Gerais 31270-901, Brazil; ‡ Laboratório de Biologia Celular, IOC, FIOCRUZ, Rio de Janeiro, Rio de Janeiro 21045-900, Brazil; § Département Médicaments et Technologies pour la Santé (DMTS), Université Paris-Saclay, CEA, INRAE, SCBM, Gif-sur-Yvette 91191, France

## Abstract

A Pd-mediated C–H activation methodology was developed for the direct arylation of quinoidal skeletons, both by means of homogeneous catalysis and heterogeneous catalysis using metal-decorated carbon nanotubes, enabling the direct functionalization of quinones in moderate to excellent yields. The derivatives obtained were tested against trypomastigote forms of *Trypanosoma cruzi*, showing promising biological activity.

## Graphical abstract

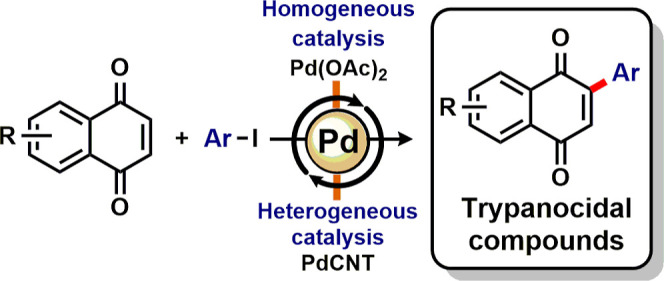

## Introduction

Quinones constitute a class of ubiquitous dicarbonyl compounds widely distributed in nature, where they are found as core structural motifs in numerous secondary metabolites, including lawsone, lapachol, and vitamin K. Beyond their structural diversity, quinones fulfill pivotal biochemical functions. Ubiquinone (coenzyme Q_10_), for example, is an essential lipid-soluble electron carrier within the mitochondrial electron transport chain, facilitating oxidative phosphorylation by shuttling electrons between complexes I/II and III. It also participates in photosynthetic electron transport in chloroplasts. Additionally, due to their redox-active nature, quinones serve as potent oxidizing agents, playing significant roles in cellular redox homeostasis and signal transduction pathways.^1^ This scaffold has attracted considerable attention from the scientific community due to its significant range of biological activities, such as anticancer,^2^ antimalarial^3^ and antimicrobial.^4^ In addition to biology and medicine, quinones are widespread in materials science,^5^ acting as luminescent sensors, and as anolytes and catholytes in redox flow batteries.^6^

The myriad of quinoidal medicinal properties is directly related to the ability of their carbonyl groups to accept one and/or two electrons to form anionic/dianion species in the cellular environment, which in turn are responsible for triggering the generation of oxidative radicals, such as hydroxyl and superoxide radicals, better known as reactive oxygen species (ROS). These radicals are key mediators of cell death-related processes.^7^ Surprisingly, this ability of quinones to act as electron carriers makes them excellent candidates for battery components^8^ and organic electronics in general.^9^ At the same time, this oxidative feature is related to the difficulty in functionalizing quinones directly through C–H bond activation.^10^ In fact, in Wacker-type processes, benzoquinone is used as a more sustainable oxidizing alternative when compared to transition metal salts.^11^

Despite the growing advances in C–H activation protocols in recent years,^12^ the use of this powerful arsenal of reactions in the formation of C–C bonds in quinones still proves to be challenging. This is mainly due to the oxidizing nature of the quinoidal core that hinder catalytic cycles requiring low-valent metal species.^13^ Of all the metals used to carry out C–H activation reactions in quinones, palladium is among the most efficient.^14^

According to the World Health Organization, neglected tropical diseases mainly affect low-income populations in more vulnerable regions around the world, with Chagas disease being one of them. This endemic disease in 21 American countries is caused by the hemoflagellate protozoan *Trypanosoma cruzi*, infecting millions of people and causing thousands of deaths annually. The available chemotherapy is limited and poses several challenges, including high toxicity and therapeutic failure, reinforcing the need to search for new active molecules. To minimize possible side effects, new strategies have been developed based on biological processes established during the parasite’s relationship with its hosts. It is well documented that reactive oxygen and nitrogen species are central players in combating *T. cruzi* in vertebrate hosts.^15^

Our research group has long been developing methodologies for functionalizing quinones through the direct activation of hydrogen atoms in both the quinonoid ring (B-ring) and the benzenoid ring (A-ring), with the aim of discovering medicinal prototypes to combat trypomastigote forms of *T. cruzi*, the protozoan parasite responsible for Chagas disease. Direct modifications have led to novel quinoidal derivatives with relevant activity against the parasite (Scheme 1A).^16^ As illustrated in Scheme 1B, numerous researchers have focused on developing catalytic methods for C–H arylation of quinones.^17^ However, these approaches often rely on complex and hard-to-access catalysts, as well as the use of expensive solvents, ligands, oxidants, or cocatalysts, which can limit their applicability in certain medicinal chemistry contexts. Building on these valuable contributions and driven by the therapeutic relevance of quinones, we report here a Pd-catalyzed C–H activation protocol for their direct modification using both homogeneous and heterogeneous catalysis (Scheme 1C). In addition to the synthetic development, we also evaluated the resulting compounds and identified several bioactive derivatives with potential trypanocidal activity.

**Scheme 1:**
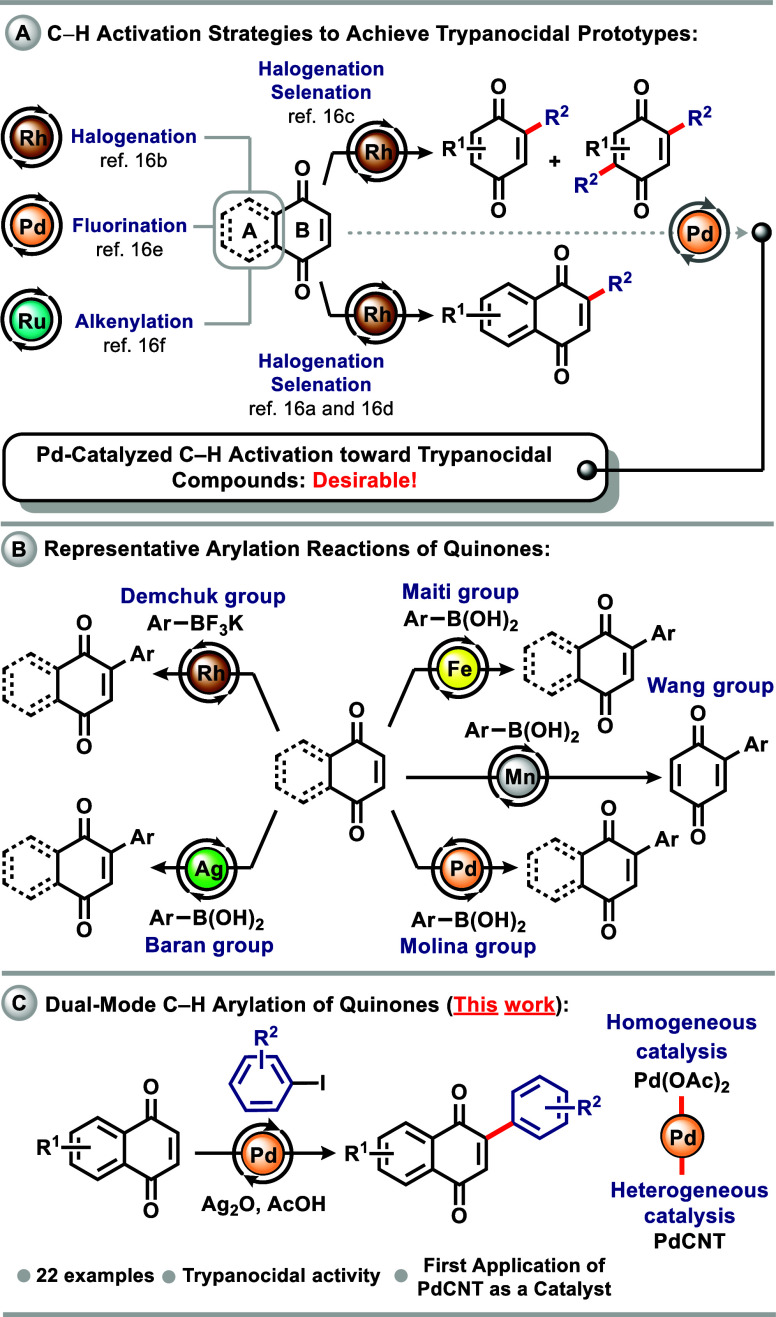
Overview

## Results and Discussion

Inspired by previous works,^17,18^ and based in part on findings by Akagi and Komatsu, who reported the use of palladium catalysis in quinone arylation,^17h^ our model reaction consisted in reacting naphthoquinone **1a** and three equivalents of iodoarene **2a** with 10 mol % of Pd­(OAc)_2_, 1 equiv of Ag_2_O, and trifluoroacetic acid (TFA) as a solvent at 120 °C for 24 h (Table 1).

**Table 1:**
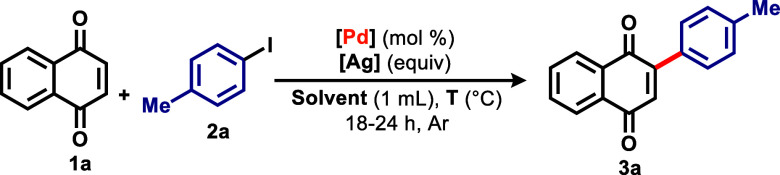
Homogeneous Catalyst Optimization^a^

entry	[Pd]-source (mol %)	[Ag] (equiv)	solvent	*T* (°C)	yield %
1	Pd(OAc)_2_ (10)	Ag_2_O (1.0)	TFA	120	68
2	Pd(OAc)_2_ (5)	Ag_2_O (1.0)	TFA	120	trace
3	Pd(OAc)_2_ (10)	Ag_2_O (1.0)	TFA	120	36^b^
4	Pd(OAc)_2_ (10)	Ag_2_O (2.0)	TFA	120	15
5		Ag_2_O (1.0)	TFA	120	NR
6	Pd(OAc)_2_ (10)		TFA	120	16
7	Pd(OAc)_2_ (10)	AgOAc (1.0)	TFA	120	49
8	Pd(OAc)_2_ (10)	Ag_2_O (1.0)	TFA	90	46
9	Pd(OAc)_2_ (10)	Ag_2_O (1.0)	TFA	150	65
10	Pd(PPh_3_)_2_Cl_2_ (10)	Ag_2_O (1.0)	TFA	120	49
11	Pd(OAc)_2_ (10)	Ag_2_O (1.0)	1,4-dioxane	120	26
12	Pd(OAc)_2_ (10)	Ag_2_O (1.0)	1,2-DCE	120	31
13	Pd(OAc)_2_ (10)	Ag_2_O (1.0)	AcOH	120	93
14	Pd(OAc)_2_ (10)	Ag_2_O (1.0)	AcOH	120	79^c^
15	Pd(OAc)_2_ (0.1)	Ag_2_O (1.0)	AcOH	120	NR^d^

a General reaction conditions: **1a** (0.2 mmol); **2a** (3.0 equiv), catalysts (0.1–10 mol %), Ag_2_O or AgOAc (1.0 equiv), solvent (1 mL), 90–150 °C, 18–24 h.

b **2a** (2.0 equiv).

c Air atmosphere.

d AcOH (0.5 mL). NR = in all cases, the starting material was recovered. The reported yields correspond to isolated products.

Under these conditions, the desired product **3a** was obtained in 68% yield (entry 1). A decrease in the catalytic charge proved inefficient, and only traces of **3a** were detected using Pd­(OAc)_2_ at 5 mol % (entry 2), just as decreasing the iodoarene equivalents to two resulted in a 36% yield (entry 3). As evidenced in entry 4, an increase to 2 equiv of Ag_2_O resulted in a 15% yield of **3a**. While the reaction without palladium did not take place (entry 5), that without Ag_2_O resulted in a low yield of 16% (entry 6). The use of another silver source (AgOAc) was fruitless as product **3a** was obtained in 49% yield only (entry 7). Moreover, a decrease/increase in temperature also proved inefficient (90 and 150 °C, entries 8 and 9).

Next, we used another source of Pd­(II) in the reaction, and **3a** was obtained in 49% by using 10 mol % of Pd­(PPh_3_)_2_Cl_2_ (entry 10). Other nonacidic solvents also proved unsuitable for the methodology, and the use of 1,4-dioxane and 1,2-DCE resulted in yields of 26 and 31%, respectively (entries 11 and 12). To our surprise, an improved yield of 93% of **3a** was observed when using acetic acid as the reaction solvent, resulting in the best conditions (entry 13). The same reaction carried out under air atmosphere proved less efficient, with **3a** obtained in 79% yield (entry 14). For comparison purposes with the heterogeneous system, the reaction was carried out using 0.5 mL of AcOH and 0.1 mol % catalyst loading (entry 15). Under those conditions, no product was observed.

With optimized reaction conditions in hand, a scope was obtained to illustrate the new methodology (Scheme 2). For substrate with methyl substituents in different positions of the aromatic ring, the reaction was less effective than it was with **2a** (93% yield of **3a**), as derivative **3b**, containing a methyl group in *meta* position, and derivative **3c**, containing methyl groups were obtained with 63 and 81% yield, respectively. When iodobenzene **2d** was used, a slight decrease in yield was observed, with derivative **3d** obtained in 86% yield. The presence of strong electron-donating methoxy groups on the ring considerably reduced the reaction yield, as exemplified by derivative **3f** (*p*-methoxy, 72% yield and *m*-methoxy, 63% yield). Surprisingly, the reaction, when carried out with iodoarenes containing highly withdrawing groups in the *para* position of the aromatic ring also resulted in lower yields, as illustrated by derivative **3g**, containing a CF_3_ group (41% yield) and derivative **3h**, containing a methyl ester group (46% yield).

**Scheme 2:**
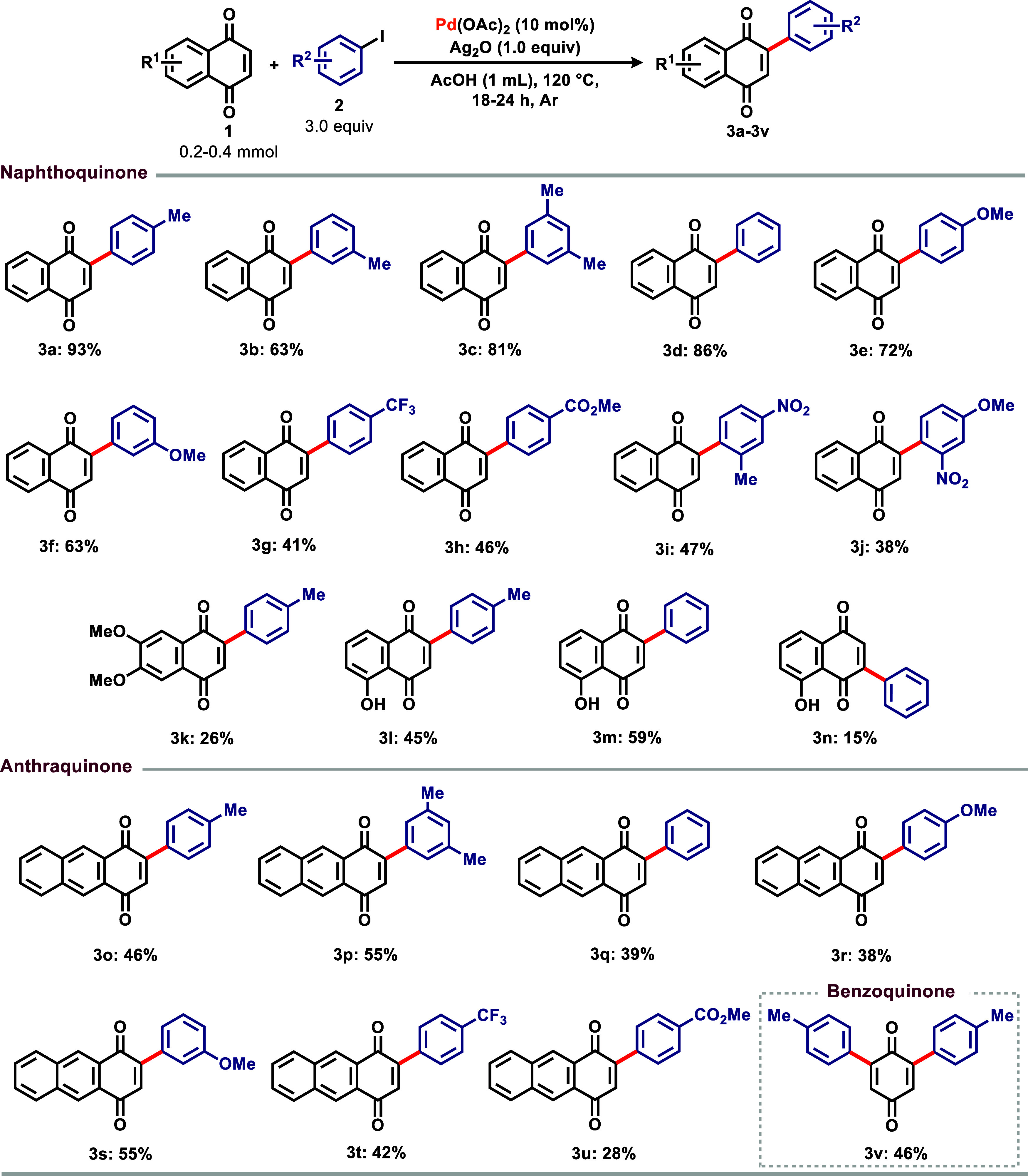
Scope for Homogeneous Catalysis

Variations containing electron-donating and electron-withdrawing groups on the iodoarene ring were tested, where lower yields were obtained, as shown with derivatives **3i** (47% yield) and **3j** (38% yield). For naphthoquinone **1b**, containing two methoxy groups on the benzenoid ring, a low yield of 26% (derivative **3k**) was observed when the reaction was carried out with **2a**. Next, juglone **1c** was subjected to reaction with **2a** and, surprisingly, only derivative **3l** was obtained in 45% yield. When performing the reaction with **2b**, a mixture of regioisomers (**3m** and **3n**) was obtained with a combined yield of 74%.

Next, 1,4-anthraquinone scaffold was also subjected to the new methodology. Contrary to the results with **1a**, both the use of iodoarenes containing electron-donating and electron-withdrawing groups led to moderate to low yields, with derivatives **3o** and **3p**, containing methyl groups in different positions of the aromatic ring being obtained in 46 and 55% yield, respectively, as well as derivatives **3r** and **3s**, containing methoxy groups in the *para* and *meta* positions, being observed in 38 and 55%. For iodoarenes containing CF_3_ and acetyl electron-withdrawing groups, moderate and low yields were obtained, as shown by derivatives **3t** and **3u**. Finally, the use of benzoquinone **1e** led to a double functionalization, with derivative **3v** being observed in 46% yield.

Aiming for a more sustainable and modern approach to the above transformation, we developed a heterogeneous and reusable catalyst based on carbon nanotubes decorated with palladium nanoparticles (PdCNT, Figure 1), prepared by a layer-by-layer protocol adapted from our previous works.^19^ Therefore, we subjected the heterogeneous system to the best reaction conditions developed above for the homogeneous process and varied the catalyst loadings (Table 2).

**Figure 1:**
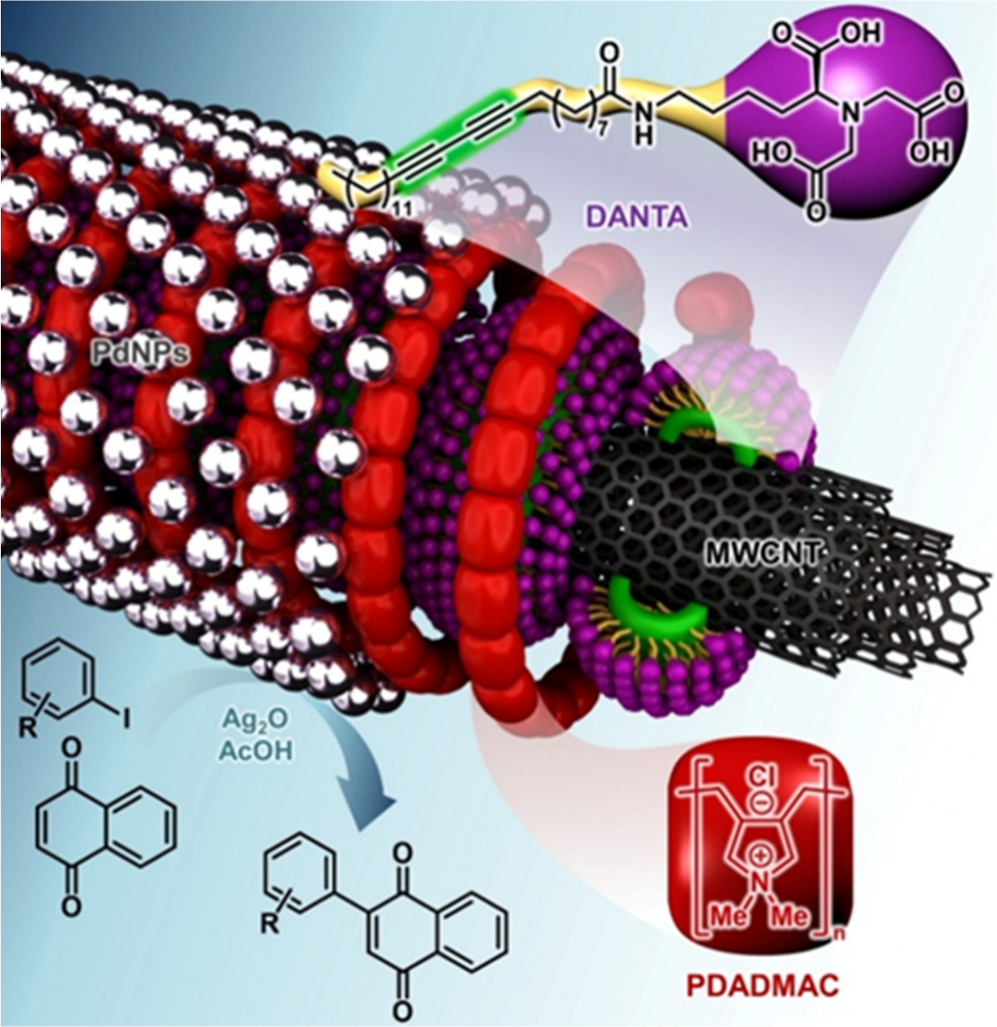
Carbon nanotube-palladium nanohybrid catalyst (PdCNT).

**Table 2:**
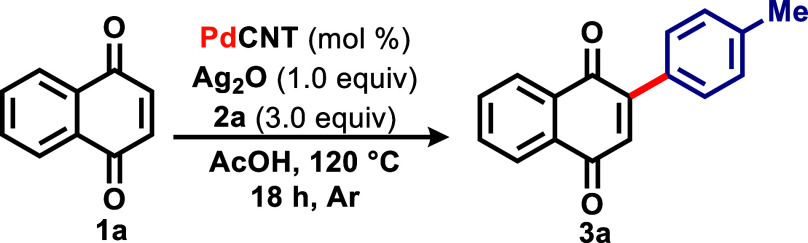
Heterogeneous Catalyst Optimization

entry	PdCNT (mol %)	Ag_2_O (equiv)	AcOH (mL)	yield %
1	0.1	1.0	1.0	60
2	0.1		1.0	trace
3	0.05	1.0	1.0	27
4	0.3	1.0	1.0	61
5	1.0	1.0	1.0	65
6	0.1	1.0	1.0	48^a^
7	0.1	1.0	0.5	79

a General reaction conditions: **1a** (0.2 mmol); **2a** (3.0 equiv), catalyst (0.05–1.0 mol %), Ag_2_O (1.0 equiv), AcOH (0.5–1.0 mL), 120 °C, 18 h. Air atmosphere. The reported yields correspond to isolated products.

C–H activation via the heterogeneous pathway proved fruitful, and **3a** was obtained in 60% yield using only 0.1 mol % of palladium (entry 1), which is 2 orders of magnitude lower than the amount of Pd used under homogeneous conditions. Next, a fine-tuning of the heterogeneous catalysis reaction conditions was carried out to optimize this process. The reaction carried out in the absence of a silver source resulted in only traces of the product, while a decrease in catalyst concentration to 0.05 mol % resulted in a decrease in yield (entry 3), and a 3-fold and a 10-fold increase in the Pd-loading caused no significant increase in yields (entries 4 and 5). The reaction carried out under air atmosphere instead of argon was detrimental to the yield (48%, entry 6). Finally, an increase in the reaction concentration resulted in the best result, with **3a** being obtained in 79% yield (entry 7).

After tuning the conditions for heterogeneous catalysis, a scope was built to demonstrate its viability (Scheme 3). Heterogeneous catalysis provided slightly lower yields compared to its homogeneous counterpart as derivative **3a** was obtained in 79% yield (vs 93% homogeneous). However, under homogeneous conditions, with 0.1 mol % catalyst and 0.5 mL of solvent, the reaction did not occur (entry 15, Table 1). Slightly lower yields were also obtained for derivatives containing methyl groups in varying positions of the aromatic ring (**3b** and **3c**, 53 and 71% yield, respectively). The reaction, when carried out using iodobenzene **2d**, yielded derivative **3d** in 74% yield, while derivatives containing methoxy groups in the *para* (**3e**) and *meta* (**3f**) positions of the aromatic ring were obtained in 60 and 52% yield. For iodoarenes containing electron-withdrawing groups, the reaction worked in low yields, with derivative **3g** being obtained in 27% yield and derivative **3h** in 35% yield.

**Scheme 3:**
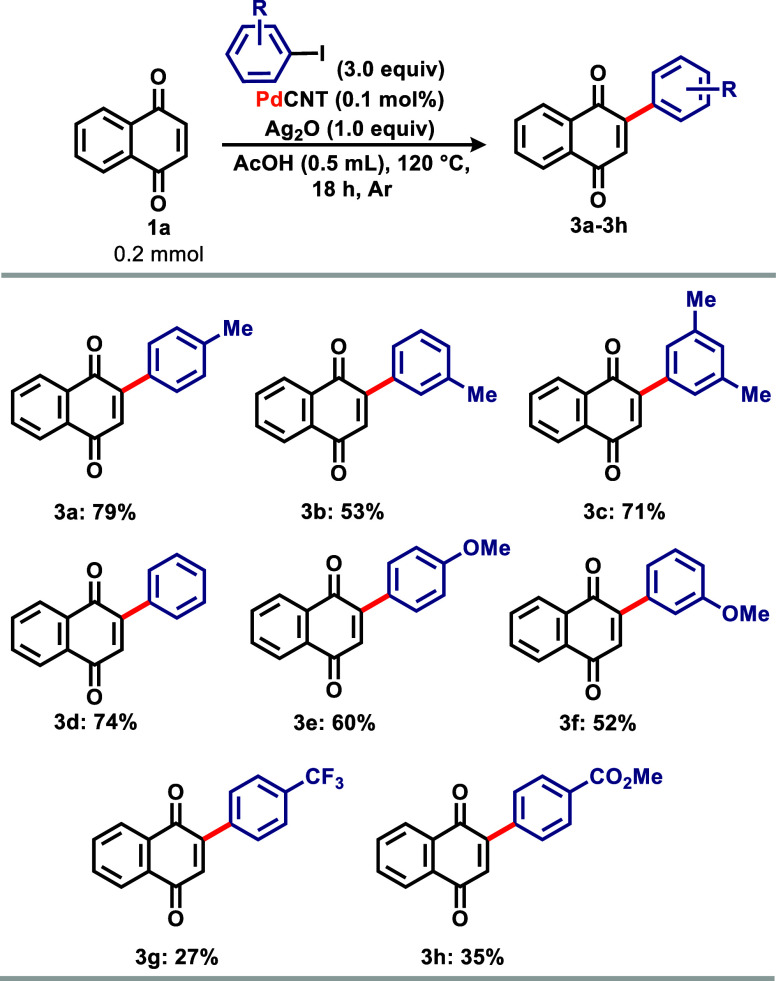
Scope for Heterogeneous Catalysis

Finally, to assess the recyclability of the PdCNT catalyst, the arylation reaction (standard reaction shown in Table 2) was performed over four consecutive cycles using a straightforward recovery protocol based on mild centrifugation. After each run, the catalyst was decanted and the supernatant containing the product was collected. Fresh reagents and solvent were added directly to the recovered catalyst. The nanocatalyst maintained good performance for the initial cycles; however, from the third reuse onward, a progressive decrease in yield was observed, approximately 20% per cycle, indicating gradual catalyst deactivation.

As part of our long-lasting efforts to develop molecules to treat Chagas disease,^20^ we decided to test the biological activity of some selected substances. Overall, out of the twenty-two compounds synthesized, 17 candidates were selected through a preliminary screening, which evaluated the potential of the new substances against trypomastigote forms of *T. cruzi*, and the most active compounds are highlighted in Table 3.

**Table 3:** Biological Assays

compound	IC_50_/24 h (μM)^a^	LC_50_/24 h (μM)	SI	IC_50_/24 h (μM)^b^	SI
**3a**	36.3 ± 2.90	24.6 ± 3.2	0.68		
**3b**	38.4 ± 1.30	14.3 ± 0.5	0.37		
**3c**	218.6 ± 13.40				
**3d**	25.2 ± 2.80	9.4 ± 2.2	0.37	2.11 ± 0.1	4.45
**3e**	47.6 ± 0.60	34 ± 0.5	0.71		
**3f**	30.7 ± 2.50	8.3 ± 1.3	0.27	3.85 ± 0.01	2.15
**3g**	30.6 ± 2.10	6.8 ± 0.2	0.22		
**3h**	45.9 ± 4.3	30.6 ± 1	0.67		
**3i**	39.5 ± 4.10	6.8 ± 0.6	0.17		
**3j**	86.1 ± 7.20				
**3k**	83.3 ± 4.8				
**3l**	14.4 ± 2.1	4.8 ± 0.9	0.33	0.31 ± 0.04	15.48
**3m**	12.7 ± 0.10	<3.9		0.34 ± 0.03	<0.30
**3n**	13.7 ± 1.10	6.4 ± 0.5	0.47	0.69 ± 0.03	9.27
**3p**	>250				
**3q**	>250				
**3v**	232.8 ± 12.40				

a Mean ± SD of at least three independent experiments, 5% of blood at 4 °C. IC_50_/24h for benznidazole in μM = 103.6 ± 0.6.^16f^

b Mean ± SD of at least three independent experiments, 0% of blood at 37 °C. IC_50_/24h for benznidazole in μM = 9.68 ± 2.3.^16b^

Regarding the anthraquinone-based derivatives, two compounds (**3p** and **3q**) showed low activity (IC_50_ = >250 μM), and similar results were observed for benzoquinone derivative **3v** (IC_50_ = 232.8 μM). To our delight, all naphthoquinoidal derivatives showed biological activity superior to the reference drug benznidazole (IC_50_ = 103.6 μM), except for **3c**, containing two methyl groups in the aryl portion (IC_50_ = 218.6 μM). Moderate activity was observed for derivative **3j**, containing an electron-donating and withdrawing group on the aryl portion (IC_50_ = 86.1 μM) and **3k**, containing two methoxy groups on the benzenoid ring (IC_50_ = 83.3 μM), followed by **3h** (IC_50_ = 45.9 μM), containing an acetate in its structure, and **3e** (IC_50_ = 47.6 μM), containing a methoxy substituent in the *para*-position of the aromatic ring.

Compounds **3a**, **3b** and **3i**, which contain methyl groups in different positions of the aromatic ring, as well as **3f**, which bears a methoxy substituent in *meta*-position, can be considered promising prototypes for combating trypomastigote forms of the parasite, with low IC_50_ values in the range of 30–40 μM. Interestingly, derivative **3d**, with no substituents on the aryl portion, was one of the most active of the naphthoquinoidal series, with IC_50_ = 25.2 μM. Finally, the most active derivatives of all were derived from substrate **1c**, all containing a hydroxyl moiety attached to the benzenoid ring (**3l**–**n**). Among the latter, **3m** stands out with the lowest IC_50_ value (12.7 μM) and can be considered a promising candidate for further studies.

Based on the results obtained with bloodstream trypomastigotes incubated at 4 °C, the five most promising compounds (**3d**, **3f**, **3l**, **3m** and **3n**) were selected for subsequent evaluation of their trypanocidal activity after 24 h of treatment at 37 °C. Among them, compounds **3n**, **3m** and **3l** showed the best activity profiles, with IC_50_ values of 0.69 μM, 0.34 μM and 0.31 μM, respectively. These results infer that the synthesized compounds were at least 14 times more active than benzimidazole (IC_50_ = 9.68 μM), tested under the same conditions.

## Conclusion

In summary, we report a new C–H bond activation methodology aimed at functionalizing the dicarbonyl ring of quinones via two routes, one homogeneous, using palladium acetate, and the other heterogeneous, employing carbon nanotubes decorated with palladium nanoparticles. In all, 22 examples with varying degrees of substitution were obtained in moderate to excellent yields to illustrate the new methodology and, of these, 17 candidates were evaluated against trypomastigote forms of *T. cruzi*. The biological tests showed promising results, with 11 molecules proving to be more active than the current reference drug, benznidazole, and further studies are being conducted with the most potent derivatives.

## Supplementary Material



## Notes

General remarks; substrate synthesis; palladium­(II)-catalyzed C–H arylation of quinoidal compounds; PdCNT-catalyzed C–H arylation of naphthoquinoidal compounds; biological experimentation; NMR spectra of final products (DOCX)
