# Supplementary material for: Palladium-Catalyzed C–H Arylation of Quinoidal Scaffolds Through Homogeneous and Heterogeneous Pathways: Advancing Toward Trypanocidal Prototypes
Source: ACS Omega. 2025 Aug 14;10(33):37898–905. doi: 10.1021/acsomega.5c05006 (PMC12391934; doi:10.1021/acsomega.5c05006)
Supplement: Supplementary file 1 [file ao5c05006_si_001.docx]

**Supplementary Information**

**Palladium-Catalyzed C–H Arylation of Quinoidal Scaffolds through Homogeneous and Heterogeneous Pathways: Advancing towards Trypanocidal Prototypes**

José M. C. Tavares Junior,^[a]^ Eduardo F. S. Guimarães,^[a]^ Mateus P. Nunes, ^[a]^ Renata G. Almeida,^[a]^ Maria H. Araujo,^[a]^ Victor F. S. Ramos,^[b]^ Rubem F. S. Menna-Barreto,^[b]^ Joel A. Tchuiteng Kouatchou,^[c]^ ^[c]^ Edmond Gravel,^[c]^* Eric Doris,^[c]^* Guilherme A. M. Jardim^[a]^ and Eufrânio N. da Silva Júnior*^[a]^

^[a]^ Departamento de Química, Instituto de Ciências Exatas, Universidade Federal de Minas Gerais, Belo Horizonte, MG 31270-901, Brazil. E-mail: eufranio@ufmg.br.

^[b]^ Laboratory of Cellular Biology, IOC, FIOCRUZ, Rio de Janeiro, RJ 21045-900, Brazil.

^[c]^ Université Paris-Saclay, CEA, INRAE, Département Médicaments et Technologies pour la Santé (DMTS), SCBM, 91191, Gif-sur-Yvette, France. E-mail: edmond.gravel@cea.fr, eric.doris@cea.fr

**Table of Contents**

General Remarks…………………………………………………………………………….……………S3

Substrate Synthesis…………………………………………………………………………..……………S3

Palladium(II)-Catalyzed C–H Arylation of Quinoidal Compounds…………..…………………………. S5

PdCNT-Catalyzed C–H Arylation of Naphthoquinoidal Compounds.…………….…………...…….... S12

Biological Experimentation………………………………………………...………………….….……. S14

NMR Spectra – Final Products..................................................................................................................S15

**General Remarks**

All reactions were conducted under an argon atmosphere in glassware dried with a 450 °C heat gun under high vacuum, with purity confirmed by ^1^H-NMR. Thin layer chromatography analyses (TLC) were performed using fluorescent-treated silica gel 60 (F254) coated aluminum plates and revealed under UV light, potassium permanganate and vanillin, followed by heating. Organic solutions were concentrated by rotary evaporation at 40 °C. Column chromatography using Aldrich brand silica gel 60 (230-240 mesh) was the technique of choice for purifications. ¹H, ¹³C NMR and ^19^F spectra were recorded on Varian Mercury VX 300, Inova-500, Inova-600, and Bruker Avance 300, Avance III 300, Avance III HD 400, Avance III 400, Avance III HD 500 and Avance NEO 600 instruments, respectively, employing CDCl_3_ using tetramethylsilane (TMS) for the ¹H NMR spectra as a reference. For the ¹³C spectra, the solvent signal was used as reference. Chemical shifts (*δ*) are reported in ppm and coupling constants (*J*) in Hertz (Hz). For the ^19^F, CFCl_3_ (trichlorofluoromethane) is often chosen as the reference compound and is assigned a chemical shift of 0 ppm by convention. The following abbreviations were used to note signal multiplicities: s - singlet; sl - singlet large; d - doublet; t - triplet; q - quartet; dd - doublet of doublets; ddd - doublet of doublet of doublets; dt - doublet of triplets; ddt - doublet of doublet of triplets; dq - doublet of quartets; m - multiplet; qu - quintet; sept - septet; td - triplet of doublets; tt - triplet of triplets. Bruker ATR FT-IR Alpha device and IR Prestige-21 Shimadzu spectrophotometer were used to obtain infrared spectra with wavelength absorbances, and main peaks were reported as absorption maxima (cm^−1^). Melting point analyses were performed on a Melting Point M-560 (BUCHI) apparatus. Melting points were reported as a range of temperatures.

**Substrate Synthesis**

**5-hydroxynaphthalene-1,4-dione (1c):** A 125 mL round-bottom flask was charged with acetic anhydride (12.6 mL, 13.3 mmol) and hydrogen peroxide (22.5 mL, 9.6 mmol). The mixture was stirred at 40 °C for 4 h. Subsequently, a solution of 1,5-dihydroxynaphthalene (2.40 g, 15.0 mmol) in dichloromethane (15 mL) and methanol (15 mL) was added, and the reaction mixture was stirred overnight at room temperature. **After completion**, **the reaction** was quenched with water (15 mL) and extracted with ethyl acetate (3 × 25 mL). The combined organic layers were dried over anhydrous MgSO₄, filtered, and concentrated under reduced pressure. The crude product was purified by flash column chromatography using hexane/ethyl acetate (8:2) as the eluent, affording a red solid (1.90 g, 73% yield). **^1^H NMR (400 MHz, CDCl_3_) *δ*:** 11.90 (s, 1H), 7.67–7.60 (m, 2H), 7.28 (dd, *J* = 7.7, 1.8 Hz, 1H), 6.96 (s, 2H). **^13^C NMR (100 MHz, CDCl_3_) *δ*:** 190.4, 184.4, 161.6, 139.7, 138.8, 136.7, 131.9, 124.6, 119.3, 115.1. Data consistent with the literature.^^[[1]](#footnote-1)^^

**Anthracene-1,4-dione** **(1d)**: To a solution of 1,4-dihydroxyanthracene-9,10-dione (2.0 g, 8.4 mmol) in methanol (40 mL), under argon atmosphere, NaBH₄ (1.28 g, 33.6 mmol) was added at 0 °C, and the resulting mixture was stirred for 1 h. Subsequently, 3 N HCl (25 mL) was slowly added at 0 °C. The resulting solid was collected by filtration and washed with water. After drying, the crude solid was recrystallized from acetone/petroleum ether to afford **1d** (1.55 g, 89% yield) as a brown solid. **^1^H NMR (400 MHz, CDCl_3_) *δ*:** 8.56 (s, 2H), 8.02 (s, 2H), 7.67 (s, 2H), 7.03 (s, 2H). **^13^C NMR (100 MHz, CDCl_3_) *δ*:** 184.8, 140.1, 134.9, 130.3, 129.7, 129.0, 128.4. Data consistent with the literature.^[[2]](#footnote-2)^

**Nitro-iodobenzene derivatives** (**2i** **and** **2j**): A 100 mL round-bottom flask was charged with the corresponding nitroaniline (2.0 mmol) in water (10 mL) and sulfuric acid (5 mL). The reaction mixture was stirred until a homogeneous solution was obtained. Subsequently, sodium nitrite (0.276 g, 4.0 mmol), dissolved in water (5 mL), was added dropwise at 0 °C. The reaction was stirred for 30 min to generate the diazonium salt. Then, potassium iodide (0.83 g, 5.0 mmol), dissolved in water (5 mL), was added dropwise at room temperature, and the reaction mixture was stirred for an additional 30 min. The resulting solid was collected by vacuum filtration and purified by silica gel column chromatography using hexane/ethyl acetate (95:5) as the eluent, affording an off-white solid (**2i**, 0.252 g, 48% yield) or an orange solid (**2j**, 0.218 g, 39% yield).

**1-iodo-2-methyl-4-nitrobenzene (2i): ^1^H NMR (400 MHz, CDCl_3_) *δ*:** 8.06 (d, *J* = 2.9 Hz, 1H), 7.99 (d, *J* = 8.6 Hz, 1H), 7.71 (dd, *J* = 8.6, 2.8 Hz, 1H), 2.54 (s, 3H). **^13^C NMR (100 MHz, CDCl_3_) *δ*:** 143.6, 140.0, 123.9, 122.0, 109.5, 28.4. Data consistent with the literature.^[[3]](#footnote-3)^

**1-iodo-4-methoxy-2-nitrobenzene (2j): ^1^H NMR (600 MHz, CDCl_3_) *δ*:** 7.86 (d, *J* = 8.8 Hz, 1H), 7.40 (d, *J* = 2.9 Hz, 1H), 6.86 (dd, *J* = 8.8, 3.0 Hz, 1H), 3.85 (s, 3H). **^13^C NMR (150 MHz, CDCl_3_) *δ*:** 160.3, 153.6, 142.2, 120.6, 111.1, 74.6, 56.1. Data consistent with the literature.^[[4]](#footnote-4)^

**Palladium(II)-Catalyzed C–H Arylation of Quinoidal Compounds**

**Scheme S1.** General reaction for the palladium(II)-catalyzed C–H arylation of quinoidal compounds.

A 5.0 mL resealable vessel was charged with the corresponding quinone (0.2–0.4 mmol), Pd(OAc)₂ (10 mol%), Ag₂O (1.0 equiv.), and the corresponding iodoarene (3.0 equiv.). The vessel was sealed with a rubber septum, and an inert argon atmosphere was achieved using the Schlenk technique. Acetic acid (1 mL) was added via syringe, and the reaction mixture was stirred at 120 °C for 18 h. After completion, the reaction was filtered through a Celite pad and washed with CH₂Cl₂ (3 × 5 mL). The combined filtrates were concentrated under reduced pressure, and the resulting residue was purified by silica gel column chromatography to afford the desired products.

**2-(*p*-tolyl)naphthalene-1,4-dione (3a):** The general procedure was followed by using **1a** (31.6 mg, 0.2 mmol), 1-iodo-4-methylbenzene (130.8 mg, 0.6 mmol), Ag_2_O (46.4 mg, 0.2 mmol) and Pd(OAc)_2_ (4.49 mg, 0.02 mmol). Purification by column chromatography on silica gel (n-hexane/dichloromethane 50:50) yielded **3a** (46.2 mg, 93%, 0.18 mmol) as a yellow solid; **m.p. (ºC):** 104.1–105.7. **IR (KBr): *ṽ* =** 1665, 1653, 1594, 1578, 1323, 1304, 1246, 775 cm^−1^. **^1^H NMR (400 MHz, CDCl_3_) *δ***: 8.13–8.06 (m, 1H), 8.06–7.98 (m, 1H), 7.73–7.65 (m, 2H), 7.41 (d, *J* = 8.2 Hz, 2H), 7.20 (d, *J* = 8.1 Hz, 2H), 6.98 (s, 1H), 2.34 (s, 3H). **^13^C NMR (100 MHz, CDCl_3_) *δ***: 185.2, 184.6, 148.1, 140.5, 134.6, 133.8, 133.8, 132.6, 132.1, 130.5, 129.4, 129.3, 127.0, 125.9, 21.4. Data consistent with the literature.^[[5]](#footnote-5)^

**2-(*m*-tolyl)naphthalene-1,4-dione (3b):** The general procedure was followed by using **1a** (31.6 mg, 0.2 mmol), 1-iodo-3-methylbenzene (77.0 μL, 0.6 mmol), Ag_2_O (46.4 mg, 0.2 mmol) and Pd(OAc)_2_ (4.49 mg, 0.02 mmol). Purification by column chromatography on silica gel (n-hexane/dichloromethane 50:50) yielded **3b** (31.3 mg, 63%, 0.12 mmol) as an orange solid; **m.p. (ºC):** 121.3–122.8. **IR (KBr): *ṽ* =** 1665, 1654, 1593, 1309, 1294, 1262, 1246, 776 cm^−1^. **^1^H NMR (600 MHz, CDCl_3_) *δ***: 8.22–8.17 (m, 1H), 8.15–8.10 (m, 1H), 7.81–7.77 (m, 2H), 7.42–7.36 (m, 3H), 7.34–7.29 (m, 1H), 7.08 (s, 1H), 2.45 (s, 3H). **^13^C NMR (150 MHz, CDCl_3_) *δ***: 185.2, 184.5, 148.3, 138.2, 135.1, 133.9, 133.8, 133.4, 132.5, 132.1, 130.9, 130.1, 128.4, 127.0, 126.6, 126.0, 21.5. Data consistent with the literature.^5^

**2-(3,5-dimethylphenyl)naphthalene-1,4-dione (3c):** The general procedure was followed by using **1a** (31.6 mg, 0.2 mmol), 1-iodo-3,5-dimethylbenzene (86.6 μL, 0.6 mmol), Ag_2_O (46.4 mg, 0.2 mmol) and Pd(OAc)_2_ (4.49 mg, 0.02 mmol). Purification by column chromatography on silica gel (n-hexane/dichloromethane 50:50) yielded **3c** (42.5 mg, 81%, 0.16 mmol) as a yellow solid; **m.p. (ºC):** 156.9–158.1. **IR (KBr): *ṽ* =** 1664, 1653, 1592, 1307, 1294, 1247, 851, 780 cm^−1^. **^1^H NMR (600 MHz, CDCl_3_) *δ***: 8.22–8.15 (m, 1H), 8.15–8.09 (m, 1H), 7.82–7.74 (m, 2H), 7.19 (s, 2H), 7.13 (s, 1H), 7.06 (s, 1H), 2.39 (s, 6H). **^13^C NMR (150 MHz, CDCl_3_) *δ***: 185.3, 184.6, 148.5, 138.1, 135.0, 133.8, 133.8, 133.4, 132.6, 132.1, 131.8, 127.2, 127.0, 126.0, 21.4. Data consistent with the literature.^5^

**2-phenylnaphthalene-1,4-dione (3d):** The general procedure was followed by using **1a** (31.6 mg, 0.2 mmol), iodobenzene (66.9 μL, 0.6 mmol), Ag_2_O (46.4 mg, 0.2 mmol) and Pd(OAc)_2_ (4.49 mg, 0.02 mmol). Purification by column chromatography on silica gel (n-hexane/dichloromethane 50:50) yielded **3d** (40.3 mg, 86%, 0.17 mmol) as a yellow solid; **m.p. (ºC):** 94.7–96.3. **IR (KBr): *ṽ* =** 1665, 1651, 1588, 1307, 1246, 759 cm^−1^. **^1^H NMR (400 MHz, CDCl_3_) *δ***: 8.24–8.16 (m, 1H), 8.18–8.09 (m, 1H), 7.85–7.76 (m, 2H), 7.68–7.51 (m, 2H), 7.58–7.41 (m, 3H), 7.09 (s, 1H). **^13^C NMR (100 MHz, CDCl_3_) *δ***: 185.2, 184.4, 148.1, 135.2, 133.9, 133.8, 133.4, 132.5, 132.1, 130.6, 130.1, 129.5, 128.5, 127.6, 127.1, 126.0. Data consistent with the literature.^5^

**2-(4-methoxyphenyl)naphthalene-1,4-dione (3e):** The general procedure was followed by using **1a** (31.6 mg, 0.2 mmol), 1-iodo-4-methoxybenzene (140.4 mg, 0.6 mmol), Ag_2_O (46.4 mg, 0.2 mmol) and Pd(OAc)_2_ (4.49 mg, 0.02 mmol). Purification by column chromatography on silica gel (n-hexane/dichloromethane 50:50) yielded **3e** (38.1 mg, 72%, 0.14 mmol) as a red solid; **m.p. (ºC):** 123.2–125.0. **IR (KBr): *ṽ* =** 1663, 1603, 1594, 1511, 1298, 1521, 778 cm^−1^. **^1^H NMR (400 MHz, CDCl_3_) *δ***: 8.11–8.04 (m, 1H), 8.05–7.97 (m, 1H), 7.71–7.63 (m, 2H), 7.49 (d, *J* = 8.9 Hz, 2H), 6.95 (s, 1H), 6.90 (d, *J* = 8.9 Hz, 2H), 3.78 (s, 3H). **^13^C NMR (100 MHz, CDCl_3_) *δ***: 185.18, 184.80, 161.34, 147.38, 133.75, 133.73, 133.71, 132.59, 132.14, 131.10, 127.01, 125.88, 125.68, 114.06, 55.41. Data consistent with the literature.^5^

**2-(3-methoxyphenyl)naphthalene-1,4-dione (3f):** The general procedure was followed by using **1a** (31.6 mg, 0.2 mmol), 1-iodo-3-methoxybenzene (71.5 μL, 0.6 mmol), Ag_2_O (46.4 mg, 0.2 mmol) and Pd(OAc)_2_ (4.49 mg, 0.02 mmol). Purification by column chromatography on silica gel (n-hexane/dichloromethane 50:50) yielded **3f** (33.3 mg, 63%, 0.12 mmol) as an orange solid; **m.p. (ºC):** 116.8–118.1. **IR (KBr): *ṽ* =** 1666, 1656, 1594, 1268, 1250, 1231, 773 cm^−1^. **^1^H NMR (400 MHz, CDCl_3_) *δ***: δ 8.23–8.15 (m, 1H), 8.17–8.06 (m, 1H), 7.83–7.74 (m, 2H), 7.39 (t, *J* = 8.0 Hz, 1H), 7.15 (dt, *J* = 7.6, 1.3 Hz, 1H), 7.13–7.09 (m, 1H), 7.08 (s, 1H), 7.03 (dd, *J* = 7.8, 3.2 Hz, 1H), 3.87 (s, 3H). **^13^C NMR (100 MHz, CDCl_3_) *δ***: 185.2, 184.3, 159.5, 148.0, 135.3, 134.7, 133.93, 133.85, 132.5, 132.1, 129.5, 127.1, 126.0, 121.9, 115.9, 114.9, 55.4. Data consistent with the literature.^5^

**2-(4-(trifluoromethyl)phenyl)naphthalene-1,4-dione (3g):** The general procedure was followed by using **1a** (31.6 mg, 0.2 mmol), 1-iodo-4-(trifluoromethyl)benzene (88.2 μL, 0.6 mmol), Ag_2_O (46.4 mg, 0.2 mmol) and Pd(OAc)_2_ (4.49 mg, 0.02 mmol). Purification by column chromatography on silica gel (n-hexane/dichloromethane 50:50) yielded **3g** (24.8 mg, 41%, 0.08 mmol) as a yellow solid; **m.p. (ºC):** 121.9–123.7. **IR (KBr): *ṽ* =** 1663, 1593, 1352, 1326, 1111, 1069, 848 cm^−1^. **^1^H NMR (600 MHz, CDCl_3_) *δ***: 8.22–8.18 (m, 1H), 8.17–8.12 (m, 1H), 7.84–7.79 (m, 2H), 7.75 (d, *J* = 8.2 Hz, 2H), 7.69 (d, *J* = 8.6 Hz, 2H), 7.11 (s, 1H). **^13^C NMR (150 MHz, CDCl_3_) *δ***: 184.8, 183.9, 146.9, 136.9 (q, *J* = 1.5 Hz), 136.1, 134.2, 134.1, 132.2, 132.0, 131.8 (q, *J* = 32.8 Hz), 129.8, 127.2, 126.2, 125.4 (q, *J* = 3.6 Hz), 123.9 (q, *J* = 272.3 Hz). **^19^F NMR (375 MHz, CDCl_3_) *δ*:** -62.87. Data consistent with the literature.^5^

**Methyl 4-(1,4-dioxo-1,4-dihydronaphthalen-2-yl)benzoate (3h):** The general procedure was followed by using **1a** (31.6 mg, 0.2 mmol), methyl 4-iodobenzoate (157.2 mg, 0.6 mmol), Ag_2_O (46.4 mg, 0.2 mmol) and Pd(OAc)_2_ (4.49 mg, 0.02 mmol). Purification by column chromatography on silica gel (n-hexane/dichloromethane 50:50) yielded **3h** (26.7 mg, 46%, 0.09 mmol) as a yellow solid; **m.p. (ºC):** 108.3–110.1. **IR (KBr): *ṽ* =** 2924, 9853, 1723, 1593, 1436, 1272, 1105, 762 cm^−1^. **^1^H NMR (400 MHz, CDCl_3_) *δ***: 8.22–8.17 (m, 1H), 8.16–8.11 (m, 3H), 7.83–7.78 (m, 2H), 7.65 (d, *J* = 8.7 Hz, 2H), 7.12 (s, 1H), 3.96 (s, 3H). **^13^C NMR (100 MHz, CDCl_3_) *δ***: 183.8, 182.9, 165.5, 146.2, 136.7, 135.0, 133.1, 133.0, 131.3, 131.0, 130.3, 128.6, 128.5, 126.1, 125.1, 51.3. Data consistent with the literature.^[[6]](#footnote-6)^

**2-(2-methyl-4-nitrophenyl)naphthalene-1,4-dione (3i):** The general procedure was followed by using **1a** (31.6 mg, 0.2 mmol), 1-iodo-2-methyl-4-nitrobenzene (157.8 mg, 0.6 mmol), Ag_2_O (46.4 mg, 0.2 mmol) and Pd(OAc)_2_ (4.49 mg, 0.02 mmol). Purification by column chromatography on silica gel (n-hexane/dichloromethane 50:50) yielded **3i** (27.6 mg, 47%, 0.09 mmol) as a brown solid; **m.p. (ºC):** 166.9–167.3. **IR (KBr): *ṽ* =** 1664, 1592, 1518, 1346, 1296, 1258, 1248, 785, 720 cm^−1^. **^1^H NMR (400 MHz, CDCl_3_) *δ***: 8.20–8.12 (m, 4H), 7.86–7.81 (m, 2H), 7.36 (d, *J* = 8.3 Hz, 1H), 6.96 (s, 1H), 2.34 (s, 3H). **^13^C NMR (100 MHz, CDCl_3_) *δ***: 184.5, 183.3, 148.6, 148.4, 140.3, 138.6, 137.3, 134.4, 134.3, 132.0, 131.9, 130.3, 127.2, 126.4, 125.1, 121.0, 20.5. **HRMS**: Calcd for [C_17_H_12_NO_4_]^+^ [M + H]^+^ 294.0766, found 294.0768.

**2-(4-methoxy-2-nitrophenyl)naphthalene-1,4-dione (3j):** The general procedure was followed by using **1a** (31.6 mg, 0.2 mmol), 1-iodo-4-methoxy-2-nitrobenzene (167.4 mg, 0.6 mmol), Ag_2_O (46.4 mg, 0.2 mmol) and Pd(OAc)_2_ (4.49 mg, 0.02 mmol). Purification by column chromatography on silica gel (n-hexane/dichloromethane 50:50) yielded **3j** (23.5 mg, 38%, 0.07 mmol) as a yellow solid; **m.p. (ºC):** 235.5 (degradation). **IR (KBr): *ṽ* =** 1665, 1528, 1307, 1294, 1029, 850, 779 cm^−1^. **^1^H NMR (400 MHz, DMSO-d_6_) *δ***: 8.09–8.01 (m, 3H), 7.93–7.90 (m, 3H), 7.74 (s, 1H), 7.17 (s, 1H), 3.80 (s, 3H). **^13^C NMR (100 MHz, DMSO-d_6_) *δ***: 184.8, 182.8, 158.0, 155.1, 145.0, 142.0, 137.8, 134.93, 134.87, 132.1, 132.0, 129.0, 127.0, 126.3, 108.9, 76.3, 57.2. **HRMS:** Calcd for [C_17_H_11_NO_5_]^+^ [M + H]^+^ 309.0632, found 309.0631.

**6,7-dimethoxy-2-(*p*-tolyl)naphthalene-1,4-dione (3k):** The general procedure was followed by using **1b** (43.6 mg, 0.2 mmol), 1-iodo-4-methylbenzene (130.8 mg, 0.6 mmol), Ag_2_O (46.4 mg, 0.2 mmol) and Pd(OAc)_2_ (4.49 mg, 0.02 mmol). Purification by column chromatography on silica gel (n-hexane/dichloromethane 50:50) yielded **3k** (16.0 mg, 26%, 0.05 mmol) as an orange solid; **m.p. (ºC):** 155.7-157.0. **IR (KBr): *ṽ* =** 1656, 1580, 1511, 1341, 1317,1303, 1041, 880, 822 cm^−1^. **^1^H NMR (400 MHz, CDCl_3_) *δ***: 7.51 (s, 1H), 7.45 (s, 1H), 7.40 (d, *J* = 8.2 Hz, 2H), 7.20 (d, *J* = 8.4 Hz, 2H), 6.88 (s, 1H), 3.97 (s, 6H), 2.34 (s, 3H). **^13^C NMR (100 MHz, CDCl_3_) *δ***: 184.8, 184.0, 153.5, 147.7, 140.2, 134.3, 130.7, 130.6, 129.4, 129.2, 128.3, 127.2, 126.9, 108.5, 107.9, 107.3, 56.5, 21.4. **HRMS**: Calcd for [C_19_H_17_O_4_]^+^ [M + H]^+^ 309.1127, found 309.1128.

**5-hydroxy-2-(*p*-tolyl)naphthalene-1,4-dione (3l):** The general procedure was followed by using **1c** (52.2 mg, 0.3 mmol), 1-iodo-4-methylbenzene (196.2 mg, 0.9 mmol), Ag_2_O (69.5 mg, 0.3 mmol) and Pd(OAc)_2_ (6.73 mg, 0.03 mmol). Purification by column chromatography on silica gel (toluene) yielded **3l** (35.7 mg, 45%, 0.13 mmol) as an orange solid; **m.p. (ºC):** 111.3–112.5. **IR (KBr): *ṽ* =** 3458, 2923, 2853, 1639, 1456, 1255, 823, 798, 763, 741, 668 cm^−1^. **^1^H NMR (400 MHz, CDCl_3_) *δ***: 12.05 (s, 1H), 7.71 (dd, *J* = 7.6, 1.3 Hz, 1H), 7.65 (t, *J* = 7.9 Hz, 1H), 7.49 (d, *J* = 8.3 Hz, 2H), 7.29 (d, *J* = 8.2 Hz, 3H), 7.03 (s, 1H), 2.43 (s, 3H). **^13^C NMR (100 MHz, CDCl_3_) *δ***: 190.3, 183.9, 161.1, 149.2, 140.9, 136.3, 134.3, 132.5, 130.2, 129.4, 129.3, 124.1, 119.7, 115.2, 21.5. Data consistent with the literature.^5^

**5-hydroxy-2-phenylnaphthalene-1,4-dione (3m):** The general procedure was followed by using **1c** (52.2 mg, 0.3 mmol), iodobenzene (100.3 μL, 0.9 mmol), Ag_2_O (69.5 mg, 0.3 mmol) and Pd(OAc)_2_ (6.73 mg, 0.03 mmol). Purification by column chromatography on silica gel (toluene) yielded **3m** (44.3 mg, 59%, 0.18 mmol) as an orange solid; **m.p. (ºC):** 116.9–118.8. **IR (KBr): *ṽ* =** 3444, 2922, 2852, 1634, 1454, 1331, 1255, 1216, 774, 749, 731, 698 cm^−1^. **^1^H NMR (400 MHz, CDCl_3_) *δ***: 12.02 (s, 1H), 7.72 (dd, *J* = 7.5, 1.3 Hz, 1H), 7.66 (t, *J* = 7.9 Hz, 1H), 7.61–7.54 (m, 2H), 7.49 (dd, *J* = 5.8, 1.8 Hz, 3H), 7.30 (dd, *J* = 8.3, 1.3 Hz, 1H), 7.05 (s, 1H). **^13^C NMR (100 MHz, CDCl_3_) *δ***: 190.2, 183.7, 161.2, 149.3, 136.4, 135.0, 133.1, 132.4, 130.3, 129.5, 128.5, 124.2, 119.8, 115.2. Data consistent with the literature.^[[7]](#footnote-7)^

**8-hydroxy-2-phenylnaphthalene-1,4-dione (3n):** obtained as a by-product of the reaction with juglone. Purification by column chromatography on silica gel (toluene) yielded **3n** (11.3 mg, 15%, 0.04 mmol) as an orange solid; **m.p. (ºC):** 124.9–126.1. **IR (KBr): *ṽ* =** 3440, 2922, 1635, 1453, 1299, 1203, 747, 697 cm^−1^. **^1^H NMR (400 MHz, CDCl_3_) *δ***: 12.17 (s, 1H), 7.74–7.64 (m, 2H), 7.60–7.54 (m, 2H), 7.50 (dd, *J* = 5.1, 2.1 Hz, 3H), 7.36–7.28 (m, 1H), 7.04 (s, 1H). **^13^C NMR (100 MHz, CDCl_3_) *δ***: 189.9, 184.3, 162.0, 148.4, 136.7, 136.3, 132.7, 132.2, 130.2, 129.4, 128.5, 124.7, 118.8, 115.3. Data consistent with the literature.^7^

**2-(*p*-tolyl)anthracene-1,4-dione (3o):** The general procedure was followed by using **1d** (41.6 mg, 0.2 mmol), 1-iodo-4-methylbenzene (130.8 mg, 0.6 mmol), Ag_2_O (46.4 mg, 0.2 mmol) and Pd(OAc)_2_ (4.49 mg, 0.02 mmol). Purification by column chromatography on silica gel (*n*-hexane/dichloromethane 50:50) yielded **3o** (27.4 mg, 46%, 0.09 mmol) as an orange solid; **m.p. (ºC):** 184.1–185.2. **IR (KBr): *ṽ* =** 1670, 1653, 1613, 1593, 1274, 1264, 1170, 758 cm^−1^. **^1^H NMR (600 MHz, CDCl_3_) *δ***: 8.69 (s, 1H), 8.62 (s, 1H), 8.11–8.02 (m, 2H), 7.69 (dd, *J* = 5.7, 3.9 Hz, 2H), 7.54 (d, *J* = 8.1 Hz, 2H), 7.31 (d, *J* = 8.3 Hz, 2H), 7.15 (s, 1H), 2.44 (s, 3H). **^13^C NMR (150 MHz, CDCl_3_) *δ***: 184.9, 184.1, 149.4, 140.5, 136.2, 135.0, 134.8, 130.8, 130.23, 130.16, 129.49, 129.46, 129.3, 129.1, 128.7, 128.2, 21.5. Data consistent with the literature.^[[8]](#footnote-8)^

**2-(3,5-dimethylphenyl)anthracene-1,4-dione (3p):** The general procedure was followed by using **1d** (41.6 mg, 0.2 mmol), 1-iodo-3,5-dimethylbenzene (86.6 μL, 0.6 mmol), Ag_2_O (46.4 mg, 0.2 mmol) and Pd(OAc)_2_ (4.49 mg, 0.02 mmol). Purification by column chromatography on silica gel (*n*-hexane/dichloromethane 50:50) yielded **3t** (34.4 mg, 55%, 0.11 mmol) as a red solid; **m.p. (ºC):** 155.9–157.3. **IR (KBr): *ṽ* =** 1669, 1655, 1617, 1590, 1456, 1284, 1265, 848, 746 cm^−1^. **^1^H NMR (400 MHz, CDCl_3_) *δ***: 8.68 (s, 1H), 8.61 (s, 1H), 8.06 (dt, *J* = 7.0, 3.9 Hz, 2H), 7.74–7.62 (m, 2H), 7.23 (s, 2H), 7.13 (s, 2H), 2.40 (s, 6H). **^13^C NMR (100 MHz, CDCl_3_) *δ***: 184.9, 184.1, 149.9, 138.0, 136.6, 135.0, 134.8, 133.6, 131.8, 130.23, 130.16, 129.48, 129.46, 129.4, 129.1, 128.7, 128.2, 127.3, 21.4. Data consistent with the literature.^8^

**2-phenylanthracene-1,4-dione (3q):** The general procedure was followed by using **1d** (41.6 mg, 0.2 mmol), iodobenzene (66.9 μL, 0.6 mmol), Ag_2_O (46.4 mg, 0.2 mmol) and Pd(OAc)_2_ (4.49 mg, 0.02 mmol). Purification by column chromatography on silica gel (*n*-hexane/dichloromethane 50:50) yielded **3q** (22.17 mg, 39%, 0.07 mmol) as an orange solid; **m.p. (ºC):** 157.7–159.6. **IR (KBr): *ṽ* =** 1666, 1614, 1271, 757, 693 cm^−1^. **^1^H NMR (600 MHz, CDCl_3_) *δ***: 8.69 (s, 1H), 8.62 (s, 1H), 8.12–8.00 (m, 2H), 7.73–7.65 (m, 2H), 7.67–7.59 (m, 2H), 7.58–7.33 (m, 3H), 7.17 (s, 1H). **^13^C NMR (150 MHz, CDCl_3_) *δ***: 184.8, 184.0, 149.5, 136.9, 135.0, 134.8, 133.7, 130.3, 130.2, 130.1, 129.6, 129.50, 129.48, 129.0, 128.7, 128.5, 128.3. Data consistent with the literature.^8^

**2-(4-methoxyphenyl)anthracene-1,4-dione (3r):** The general procedure was followed by using **1d** (41.6 mg, 0.2 mmol), 1-iodo-4-methoxybenzene (140.4 mg, 0.6 mmol), Ag_2_O (46.4 mg, 0.2 mmol) and Pd(OAc)_2_ (4.49 mg, 0.02 mmol). Purification by column chromatography on silica gel (*n*-hexane/dichloromethane 50:50) yielded **3r** (22.2 mg, 39%, 0.07 mmol) as an orange solid; **m.p. (ºC):** 214.8–216.1. **IR (KBr): *ṽ* =** 1671, 1651, 1588, 1512, 1299, 1258, 826, 749 cm^−1^. **^1^H NMR (400 MHz, CDCl_3_) *δ***: 8.70 (s, 1H), 8.63 (s, 1H), 8.10–8.06 (m, 2H), 7.71–7.67 (m, 2H), 7.64 (d, *J* = 8.9 Hz, 2H), 7.15 (s, 1H), 7.02 (d, *J* = 8.9 Hz, 2H), 3.89 (s, 3H). **^13^C NMR (100 MHz, CDCl_3_) *δ***: 184.9, 184.4, 161.4, 148.8, 140.1, 135.4, 135.0, 134.9, 131.2, 130.23, 130.15, 129.6, 129.5, 129.4, 129.2, 128.9, 128.8, 128.1, 126.0, 114.1, 55.4. Data consistent with the literature.^8^

**2-(3-methoxyphenyl)anthracene-1,4-dione (3s):** The general procedure was followed by using **1d** (41.6 mg, 0.2 mmol), 1-iodo-3-methoxybenzene (71.5 μL, 0.6 mmol), Ag_2_O (46.4 mg, 0.2 mmol) and Pd(OAc)_2_ (4.49 mg, 0.02 mmol). Purification by column chromatography on silica gel (*n*-hexane/dichloromethane 50:50) yielded **3s** (22.2 mg, 39%, 0.07 mmol) as an orange solid; **m.p. (ºC):** 184.4–185.1. **IR (KBr): *ṽ* =** 1654, 1599, 1282, 1048, 868, 782, 754, 743, 684 cm^−1^. **^1^H NMR (400 MHz, CDCl_3_) *δ***: 8.71 (s, 1H), 8.64 (s, 1H), 8.11 – 8.05 (m, 2H), 7.75 – 7.66 (m, 2H), 7.41 (t, *J* = 7.9 Hz, 1H), 7.20 (ddd, *J* = 7.7, 1.6, 1.0 Hz, 1H), 7.16–7.13 (m, 2H), 7.05 (ddd, *J* = 8.3, 2.6, 1.0 Hz, 1H), 3.88 (s, 3H). **^13^C NMR (100 MHz, CDCl_3_) *δ***: 184.8, 183.9, 159.5, 149.4, 137.0, 135.01, 134.97, 134.8, 130.3, 130.2, 129.59, 129.57, 129.53, 129.50, 129.0, 128.7, 128.3, 121.9, 115.9, 114.9, 55.4. **HRMS**: Calcd for [C_21_H_15_O_3_]^+^ [M + H]^+^ 315.1021, found 315.1024.

**2-(4-(trifluoromethyl)phenyl)anthracene-1,4-dione (3t):** The general procedure was followed by using **1d** (41.6 mg, 0.2 mmol), 1-iodo-4-(trifluoromethyl)benzene (88.2 μL, 0.6 mmol), Ag_2_O (46.4 mg, 0.2 mmol) and Pd(OAc)_2_ (4.49 mg, 0.02 mmol). Purification by column chromatography on silica gel (*n*-hexane/dichloromethane 50:50) yielded **3t** (29.6 mg, 42%, 0.08 mmol) as an orange solid; **m.p. (ºC):** 211.9–213.4. **IR (KBr): *ṽ* =** 1664, 1614, 1330, 1118, 1065, 849, 757 cm^−1^. **^1^H NMR (400 MHz, CDCl_3_) *δ***: 8.73 (s, 1H), 8.67 (s, 1H), 8.10 (dt, *J* = 6.7, 3.2 Hz, 2H), 7.74 (dd, *J* = 9.6, 3.6 Hz, 6H), 7.20 (s, 1H). **^13^C NMR (100 MHz, CDCl_3_) *δ***: 184.5, 183.5, 148.2, 137.7, 137.2 (q, *J* = 1.3 Hz), 135.0, 134.9, 132.0, 131.6, 130.8, 130.32, 130.27, 129.85, 129.89, 129.7, 128.7, 128.6, 128.5, 125.4 (q, *J* = 3.7 Hz), 124.1 (q, *J* = 273.8 Hz). **^19^F NMR (376 MHz, CDCl_3_) *δ*:** −62.85. Data consistent with the literature.^8^

**Methyl 4-(1,4-dioxo-1,4-dihydroanthracen-2-yl)benzoate (3u):** The general procedure was followed by using **1d** (41.6 mg, 0.2 mmol), methyl 4-iodobenzoate (157.2 mg, 0.6 mmol), Ag_2_O (46.4 mg, 0.2 mmol) and Pd(OAc)_2_ (4.49 mg, 0.02 mmol). Purification by column chromatography on silica gel (*n*-hexane/dichloromethane 50:50) yielded **3u** (19.2 mg, 28%, 0.05 mmol) as a yellow solid; **m.p. (ºC):** 210.5 (degradation). **IR (KBr): *ṽ* =** 1720, 1661, 1278, 1106, 754, 475 cm^−1^. **^1^H NMR (400 MHz, CDCl_3_) *δ***: 8.73 (s, 1H), 8.67 (s, 1H), 8.16 (d, *J* = 8.6 Hz, 2H), 8.10 (dt, *J* = 6.2, 2.9 Hz, 2H), 7.76–7.67 (m, 4H), 7.21 (s, 1H), 3.97 (s, 3H). **^13^C NMR (100 MHz, CDCl_3_) *δ***: 184.5, 183.6, 166.6, 148.6, 138.1, 137.6, 135.0, 134.9, 131.3, 130.3, 130.3, 129.8, 129.7, 129.6, 129.5, 128.8, 128.6, 128.5, 52.4. **HRMS**: Calcd for [C_22_H_15_O_4_]^+^ [M + H]^+^ 343.0970, found 343.0970.

**4,4''-dimethyl-[1,1':3',1''-terphenyl]-2',5'-dione (3v):** The general procedure was followed by using **1e** (41.6 mg, 0.2 mmol), 1-iodo-4-methylbenzene (261.6 mg, 1.2 mmol), Ag_2_O (46.4 mg, 0.2 mmol) and Pd(OAc)_2_ (4.49 mg, 0.02 mmol). Purification by column chromatography on silica gel (*n*-hexane/dichloromethane 50:50) yielded **3v** (26.5 mg, 46%, 0.09 mmol) as an orange solid; **m.p. (ºC):** 133.3–135.1. **IR (KBr): *ṽ* =** 1720, 1661, 1278, 1106, 754, 475 cm^−1^. **^1^H NMR (400 MHz, CDCl_3_) *δ***: 8.73 (s, 1H), 8.67 (s, 1H), 8.16 (d, *J* = 8.6 Hz, 2H), 8.10 (dt, *J* = 6.2, 2.9 Hz, 2H), 7.76–7.67 (m, 4H), 7.21 (s, 1H), 3.97 (s, 3H). **^13^C NMR (100 MHz, CDCl_3_) *δ***: 187.8, 186.5, 146.5, 140.4, 132.0, 130.4, 129.4, 129.3, 21.4. Data consistent with the literature.^[[9]](#footnote-9)^

**PdCNT-Catalyzed C–H Arylation of Naphthoquinoidal Compounds**

**Scheme S2.** General reaction for the PdCNT-catalyzed C–H arylation of naphthoquinoidal compounds.

PdCNT (167 μL of an aqueous suspension, [Pd] = 1.2 mM, 0.1 mol%) was added to acetic acid (0.5 mL) in an Eppendorf tube, and the mixture was centrifuged (5000 × g, 6 min). The supernatant was discarded, and the pelleted catalyst was dried by slow purging with nitrogen gas. A 5.0 mL resealable vessel was charged with **1a** (0.2 mmol), Ag₂O (1.0 equiv., 0.2 mmol), and the corresponding iodoarene (3.0 equiv., 0.6 mmol). The vessel was sealed with a rubber septum, and an inert argon atmosphere was achieved using the Schlenk technique. Acetic acid (0.5 mL) and PdCNT (167 μL of an aqueous suspension, [Pd] = 1.2 mM, 0.1 mol%) were added via syringe. After completion of the reaction, CH₂Cl₂ (0.5 mL) was added, and the mixture was centrifuged (2000 × g, 5 min). The supernatant was collected, and the pelleted catalyst was washed twice with ethanol (2 × 2 mL) by centrifugation/resuspension. The collected organic phases were concentrated under reduced pressure and purified by silica gel column chromatography to afford the desired products.

**2-(*p*-tolyl)naphthalene-1,4-dione (3a):** The general procedure was followed by using **1a** (31.6 mg, 0.2 mmol), 1-iodo-4-methylbenzene (130.8 mg, 0.6 mmol), Ag_2_O (46.4 mg, 0.2 mmol) and PdCNT (167 μL of an aqueous suspension, [Pd] = 1.2 mM, 0.1 mol%). Purification by column chromatography on silica gel (n-hexane/dichloromethane 50:50) yielded **3a** (46.2 mg, 93%, 0.18 mmol) as a yellow solid.

**2-(*m*-tolyl)naphthalene-1,4-dione (3b):** The general procedure was followed by using **1a** (31.6 mg, 0.2 mmol), 1-iodo-3-methylbenzene (77.0 μL, 0.6 mmol), Ag_2_O (46.4 mg, 0.2 mmol) and PdCNT (167 μL of an aqueous suspension, [Pd] = 1.2 mM, 0.1 mol%). Purification by column chromatography on silica gel (n-hexane/dichloromethane 50:50) yielded **3b** (26.3 mg, 53%, 0.1 mmol) as an orange solid.

**2-(3,5-dimethylphenyl)naphthalene-1,4-dione (3c):** The general procedure was followed by using **1a** (31.6 mg, 0.2 mmol), 1-iodo-3,5-dimethylbenzene (86.6 μL, 0.6 mmol), Ag_2_O (46.4 mg, 0.2 mmol) and PdCNT (167 μL of an aqueous suspension, [Pd] = 1.2 mM, 0.1 mol%). Purification by column chromatography on silica gel (n-hexane/dichloromethane 50:50) yielded **3c** (37.2 mg, 71%, 0.14 mmol) as a yellow solid.

**2-phenylnaphthalene-1,4-dione (3d):** The general procedure was followed by using **1a** (31.6 mg, 0.2 mmol), iodobenzene (66.9 μL, 0.6 mmol), Ag_2_O (46.4 mg, 0.2 mmol) and PdCNT (167 μL of an aqueous suspension, [Pd] = 1.2 mM, 0.1 mol%). Purification by column chromatography on silica gel (n-hexane/dichloromethane 50:50) yielded **3d** (40.3 mg, 86%, 0.17 mmol) as a yellow solid.

**2-(4-methoxyphenyl)naphthalene-1,4-dione (3e):** The general procedure was followed by using **1a** (31.6 mg, 0.2 mmol), 1-iodo-4-methoxybenzene (140.4 mg, 0.6 mmol), Ag_2_O (46.4 mg, 0.2 mmol) and PdCNT (167 μL of an aqueous suspension, [Pd] = 1.2 mM, 0.1 mol%Purification by column chromatography on silica gel (n-hexane/dichloromethane 50:50) yielded **3e** (31.7 mg, 60%, 0.12 mmol) as a red solid.

**2-(3-methoxyphenyl)naphthalene-1,4-dione (3f):** The general procedure was followed by using **1a** (31.6 mg, 0.2 mmol), 1-iodo-3-methoxybenzene (71.5 μL, 0.6 mmol), Ag_2_O (46.4 mg, 0.2 mmol) and PdCNT (167 μL of an aqueous suspension, [Pd] = 1.2 mM, 0.1 mol%Purification by column chromatography on silica gel (n-hexane/dichloromethane 50:50) yielded **3f** (27.5 mg, 52%, 0.1 mmol) as an orange solid.

**2-(4-(trifluoromethyl)phenyl)naphthalene-1,4-dione (3g):** The general procedure was followed by using **1a** (31.6 mg, 0.2 mmol), 1-iodo-4-(trifluoromethyl)benzene (88.2 μL, 0.6 mmol), Ag_2_O (46.4 mg, 0.2 mmol) and PdCNT (167 μL of an aqueous suspension, [Pd] = 1.2 mM, 0.1 mol%). Purification by column chromatography on silica gel (n-hexane/dichloromethane 50:50) yielded **3g** (16.3 mg, 27%, 0.05 mmol) as a yellow solid.

**Methyl 4-(1,4-dioxo-1,4-dihydronaphthalen-2-yl)benzoate (3h):** The general procedure was followed by using **1a** (31.6 mg, 0.2 mmol), methyl 4-iodobenzoate (157.2 mg, 0.6 mmol), Ag_2_O (46.4 mg, 0.2 mmol) and PdCNT (167 μL of an aqueous suspension, [Pd] = 1.2 mM, 0.1 mol%). Purification by column chromatography on silica gel (n-hexane/dichloromethane 50:50) yielded **3h** (20.3 mg, 35%, 0.07 mmol) as a yellow solid.

**Biological Experimentation**

**Animals:** All animal procedures were performed in accordance with the Guidelines for Care and Use of the Laboratory Animals of Oswaldo Cruz Institute (CEUA/IOC) and approved by the Animal Ethics Committee under the license L002/2024 of the Ethics Committee for Animal Use of the Oswaldo Cruz Institute. All experiments dealing with animals were also performed in accordance with the Brazilian Law 11.794/2008. The Albino Swiss mice were housed at a maximum of 6 individuals per cage, kept in a specific-pathogen-free (SPF) room at 20 to 22 °C under a 12/12 h light/dark cycle, 50 to 60% humidity and provided sterilized water and chow *ad libitum*.

**Trypanocidal Assays**

**5% of blood at 4 °C**: For all the experiments, stock solutions of the compounds were prepared in dimethyl sulfoxide, with the final concentration of the solvent never exceeding 0.5%, concentration known to exert no toxicity to the parasite or host cells.^[[10]](#footnote-10)^ Bloodstream trypomastigotes of Y strain^[[11]](#footnote-11)^ were obtained from infected Swiss Webster mice at the peak of parasitemia by differential centrifugation process. The parasites (5 × 10^6^ parasites per mL) plus 5% of blood were incubated at 4 °C for 24 h in 5% CO_2_ atmosphere in absence or presence of the compounds. Parasite counts were performed by light microscopy and the activity of the compounds was expressed as IC_50_/24 h, corresponding to the concentration that led to 50% lysis of the parasites. At least three independent experiments were performed, and the mean and standard deviation were calculated. The standard drug Bz was used as control.

**0% of blood at 37 °C**: The experiments were performed with the Y strain of *T. cruzi*.^11^ Stock solutions of the compounds were prepared in dimethylsulfoxide (DMSO), with the final concentration of the latter in the experiments never exceeding 0.1%. Preliminary experiments showed that at concentrations of up to 0.5%, DMSO has no deleterious effect on the parasites. Bloodstream trypomastigotes were obtained from infected Albino Swiss mice at the peak of parasitemia by differential centrifugation. The parasites were resuspended to a concentration of 10 × 10^6^ cells/mL in DMES medium. This suspension (100 µL) was added to the same volume of each of the compounds, which had been previously prepared at twice the desired final concentrations. The incubation was performed in 96-well microplates (Nunc Inc., Rochester, USA) at 37 ºC for 24 h. Benznidazole (Lafepe, Brazil), the standard drug for treatment of chagasic patients, was used as control. Cell counts were performed in a Neubauer chamber, and the activity of the compounds corresponding to the concentration that led to 50% lysis of the parasites was expressed as the IC_50_/24 h.

**NMR Spectra**

**Figure S1.** ^1^H NMR spectrum of compound **3a**.

**Figure S2.** ^13^C NMR spectrum of compound **3a**.

**Figure S3.** ^1^H NMR spectrum of compound **3b**.

**Figure S4.** ^13^C NMR spectrum of compound **3b**.

**Figure S5.** ^1^H NMR spectrum of compound **3c**.

**Figure S6.** ^13^C NMR spectrum of compound **3c**.

**Figure S7.** ^1^H NMR spectrum of compound **3d**.

**Figure S8.** ^13^C NMR spectrum of compound **3d**.

**Figure S9.** ^1^H NMR spectrum of compound **3e**.

**Figure S10.** ^13^C NMR spectrum of compound **3e**.

**Figure S11.** ^1^H NMR spectrum of compound **3f**.

**Figure S12.** ^13^C NMR spectrum of compound **3f**.

**Figure S13.** ^1^H NMR spectrum of compound **3g**.

**Figure S14.** ^13^C NMR spectrum of compound **3g**.

**Figure S15.** ^19^F NMR spectrum of compound **3g**.

**Figure S16.** ^1^H NMR spectrum of compound **3h**.

**Figure S17.** ^13^C NMR spectrum of compound **3h**.

**Figure S18.** ^1^H NMR spectrum of compound **3i**.

**Figure S19.** ^13^C NMR spectrum of compound **3i**.

**Figure S20.** ^1^H NMR spectrum of compound **3j**.

**Figure S21.** ^13^C NMR spectrum of compound **3j**.

**Figure S22.** ^1^H NMR spectrum of compound **3k**.

**Figure S23.** ^13^C NMR spectrum of compound **3k**.

**Figure S24.** ^1^H NMR spectrum of compound **3l**.

**Figure S25.** ^13^C NMR spectrum of compound **3l**.

**Figure S26.** ^1^H NMR spectrum of compound **3m**.

**Figure S27.** ^13^C NMR spectrum of compound **3m**.

**Figure S28.** ^1^H NMR spectrum of compound **3n**.

**Figure S29.** ^13^C NMR spectrum of compound **3n**.

**Figure S30.** ^1^H NMR spectrum of compound **3o**.

**Figure S31.** ^13^C NMR spectrum of compound **3o**.

**Figure S32.** ^1^H NMR spectrum of compound **3p**.

**Figure S33.** ^13^C NMR spectrum of compound **3p**.

**Figure S34.** ^1^H NMR spectrum of compound **3q**.

**Figure S35.** ^13^C NMR spectrum of compound **3q**.

**Figure S36.** ^1^H NMR spectrum of compound **3r**.

**Figure S37.** ^13^C NMR spectrum of compound **3r**.

**Figure S38.** ^1^H NMR spectrum of compound **3s**.

**Figure S39.** ^13^C NMR spectrum of compound **3s**.

**Figure S40.** ^1^H NMR spectrum of compound **3t**.

**Figure S41.** ^13^C NMR spectrum of compound **3t**.

**Figure S42.** ^19^F NMR spectrum of compound **3t**.

**Figure S43.** ^1^H NMR spectrum of compound **3u**.

**Figure S44.** ^13^C NMR spectrum of compound **3u**.

**Figure S45.** ^1^H NMR spectrum of compound **3v**.

**Figure S46.** ^13^C NMR spectrum of compound **3v**.

1. ^.^ Cui, J., Jia, J., *Eur. J. Med. Chem.*, **2021**, *225*, 113789-113796. [↑](#footnote-ref-1)
2. ^.^ Dong, Y., Peng, Z., Chen, L., Lin, Y., Lan, M., Wu, C., Shi, Z., Deng, G., He, B., *Tetrahedron*, **2024**, *155*, 133916-133921. [↑](#footnote-ref-2)
3. ^.^ Stone, I. B., Starr, R. L., Hoffmann, N., Wang, X., Evans, A. M., Nuckolls, C., Lambert, T. H., Sterigerwald, M. L., Berkelback, T. C., Roy, X., Venkataraman, L., *Chem. Sci.*, **2022**, *13*, 10798-10805. [↑](#footnote-ref-3)
4. ^.^ Ali, A. R., Hu, L., *Asian J. Org. Chem.*, **2024**, *14*, e202400421. [↑](#footnote-ref-4)
5. ^.^ Gontijo, T. B., Carvalho, R. L., Dantas-Pereiras, L., Menna-Barreto, R. F. S., Rogge, R., Ackermann, L., da Silva Júnior, E. N. *Bioorg. Med. Chem.*, **2021**, *40*, 116164-116174. [↑](#footnote-ref-5)
6. ^.^ Wang, X., Ye, Y., Ji, G., Xu, Y., Zhang, S., Feng, J., Zhang, Y., Wang, J., *Org. Lett.*, **2013**, *15*, 3730-3733. [↑](#footnote-ref-6)
7. ^.^ (a) Molina, M. T., Navarro, C., Moreno, A., Csákÿ, A. G., *Org. Lett.*, **2009**, *11*, 4938-4941. (b) Laugraud, S., Guingant, A., Chassagnard, C., D’Angelo, J., *J. Org. Chem.*, **1988**, *53*, 1557-1560. [↑](#footnote-ref-7)
8. ^.^ (a) Guha, S., Prabakar, T., Sem, S., *J. Org. Chem.*, 2022, *87*, 15421-15434. (b) Demchuk, O. M., Pietrusiewicz, K. M., *Synlett*, **2009**, *7*, 1149-1153. [↑](#footnote-ref-8)
9. ^.^ Rao, M. L. N., Giri, S., *RSC Adv.*, **2012**, *2*, 12739-12750. [↑](#footnote-ref-9)
10. J. M. Wood, N. S. Satam, V. S. Cristani, D. P. Lima, L. Dantas-Pereira, K. Salomão, R. F. S. Menna-Barreto, I. N. N.

    Namboothiri, J. F. Bower and E. N. da Silva Júnior, *Bioorg. Med. Chem.*, **2020**, *28*, 115565. [↑](#footnote-ref-10)
11. L. H. P. da Silva and V. Nussenszweig, *Folia Clin. Biol.*, **1953**, *20*, 191-207. [↑](#footnote-ref-11)
